# Supplementary material for: Facultative CTCF sites moderate mammary super-enhancer activity and regulate juxtaposed gene in non-mammary cells
Source: Nat Commun. 2017 Jul 17;8:16069. doi: 10.1038/ncomms16069 (PMC5520053; doi:10.1038/ncomms16069)
Supplement: Supplementary Information [file ncomms16069-s1.pdf]

Title of file for HTML: Supplementary Information
Description: Supplementary Tables and Supplementary Notes

Title of file for HTML: Peer Review File
Description:

Supplementary Note 1: Mutant mice used in this study. Genomic sequences are given, with CTCF binding sites highlighted red, and **deletions bolded and underlined.** **CTCF binding motifs are highlighted in blue.**

Line ΔAB
sgRNA used: N/A
Deletion size: 1,711 bp
LoxP site in Yellow

TAGAATGAAGTTCCTATACTTTCTAGAGAATAGGAACTTCGTTCGAACATAACTTCGTATAGCATACATTATACGAA
GTTATGGTACCTGCAGAATTCATGCATAAGCTTGGATCTGATGCCCTCTTCTGTTTGAAGACAGCTACAGTGTACTC
ACATACATAAAATAAGTAAATAATTTTTAAAAGGAAGGAAGGAAAGAAAGAAGGAAGAGAAATAATTTCACTTCCAT
TTGGGACTCAGAAAAAGTATAGATATCTGTCAAATAGAAAAACACAAATTTGGTAAAAGAACAAAAAAAAAATTGTT
CTTCTCATACACTGAAAAAAAAAAAAGATGTTTCTAGATGACTTTAACAAGGAGAAAAGACCCATGTGAATGGGAGGG
AAGGGGTGGAGAACACCATTCTGGGGACTGGAATACCAAAATAAATAAAGAGAAGAAAGTCAAAGCCAGGCATGGTG
GCACACATATTATTATTATTATTATTATTATTATTAATTTAAAGTATGACTGCTCTGATGCATATACACCCTCAGGC
CAGAAGAGGGCACCAGTTCCCTTATAGATGGTTGTGAGCCACCATGTAGTTGCAGGAATTGAACTTAGGACCTGTGG
AAGAGCAGCCAGTTCTCTTAACCTCTGAGCCATCTCACACACATCTTTAATTACATCATGCAAAAGGCAGAGGCAGG
AGGATCTCTGAGTCTGAGGCCAGCCAGTGAGCTCCAGGGCAGCCAGGACTGCTCTAAAGACTGTCTCTAACCCTCTC
CCCCCACCCTGCAAAAAAAAAATAAAAAATATAGCTGACCATCAACATTCATTTCTCTCTGCTTCCTGACTATGCCT
TTTGGGTCTGAGGTGAGGAGGTGCAGTAGCTCTGCTGGCTACTGTGTGCTCAGGTGGCTCAGCAGGTCACCTGTGCA
TGGAGCTCAAGGCCAGTTCAGTTTCAGCATCCACACCATTCACGTTAGGACCAGTGGTCCTTCTTCCATTCCCTCAA
GTCACTGCAGGACTAGGCACCTCCTCCCCACTGAGGCCAGATGAGGCAACCCAGTTAGGGGAACAATATCCACAGGC
AGGCAACAGAGTCAGGGTAAGCCTTAAGAAGCATTCTTGGGGCTGGGGAGATGGCTCAGTGGTTAAGAGCACCGACT
GCTCTTCCAGAGGTCCTGAGTTCAATTCCCAGCAACCACATGGTGGCTCTCAACCATCTGTAATGGAGTTTGATGCC
CTCTTCTGGTGTGTCTGAAGACAACTACAGTGTACTCATATACATTAAATAAATAAATCTTAAAAAAAAAAAGCATT
CTTTACAGCACGTTGGTCAATACAATGCCCATGTCCAGATTACAACCTGATCCTGGTCACAGAACTCAGCAGCATCT
AAGCATATCTTGCACAAGACCAACCCTAGCAATGGCCTCTGCTCCAGTTTCTGCCTTGAGCTCCTGTCCTTTCTTTG
ATGATGATGTGTGATGTGAGTCAAGTAAACCACAAGTTGCTTTGGCTATGGTTTTTATCACAGTGATAGCAACCTAA
CAAAGACATTGGCCAAAGTCCATCTTAAATCCCCTAACACTTTGGGGTTCCTTGCTCCTTTCCTAAAGCTCTACTCC
CCAGCACAAGGTTGCCTGACAAACATGGTGGCTGACCCTATCTGAGCTGCATGGCTCTAGCTCCCGGCCACATGGAT
GCCTGCCACCCGGTGGCTAACCTGTGAACTGCCCAGCTCAAGCTGCAGGGCTATTCTCCAGTCCCCAGGCAGCTTTG
AGGTTTATGGCTGACCGGCCCCGGGGTTTTCTTTTTGAACTCTAACTGTCCTAGGGCCCATAACTTCGTATAGCATA
CATTATACGAAGTTATAGTACTTCCATTTGTGTGTATGTCCTAAATTATTTTATTAATAACCCTAAGGAAGAACCCT

Line ΔC
sgRNA used: C1
Deletion size: 58 bp

TGATGAGCGGCCCTGACACCTCAAAGCCGCTTTGCTCCTTTGCACATGGATCTCAGAAAAAGCCTCTGAGTTTCCCA
TATGAACTCAGGAGACCCAACCCAACCCAACCCAACCCAACCCAACCCAACCCAACCCAACCCAACAAACACCCAAA
CCGAGAGAAATAACAAAAC**GGCTCACAGCTCCCTCCACTGGCCCATATGAGTTACTGCAGCTGCTTTATCAAAAAAC**
AGCTTCTGGGTCCACAGCAGAGCCAGGCTTTCTTCCAACCTTTCTAGGTCAAAGCCAAGTCCTGAGCTCCCAGTTCT
TTTCCTTGCTTTCTCCACGATACATGTCATCCCTGTTGACCCAGCTTGTTCAGCCACACCGGTTTCCTGATGGCCC

Line ΔD
sgRNA used: D1
Deletion size: 134 bp

CTTCCTGGAACTCACTCTGTAGACCAGGCTGGCCTCAAACCCAGAAACCCACCTGCCTCTGCCTCCCAAGTGCTGGG
ATTAAAGGTGTGTGCCACCACTGCCCGGCCCAGGTTTGTTTTTCAGGACCCTCTTGGTGGCTCACAACTTTCTGGAA
CTCATAGTTGCAAGGGGATCT**GACATCCTCTTCTGGTGGGCACAGGTACTGCATGCATGTGGTACACTGTCATACAT**
GTAGACAAATCAACCAGATACATAAGTTAGAAATGAAGAAACTGGGCTGGTGAGATGGCTCAGTGGGTAAGAGTACC
CGACTGCTCTTCCGAAGGTCTGAAGTTCAAATCCCAGCAACCACATGGTGGCTCACAACCACCTGTAATGAGATCTG

Line ΔE
sgRNA used: E1/2/3
Deletion size: 160 bp

AGAAAGGCTGTCTGTCTGAGACAATAGGCCAGTTTCCCTGACTTGGTCATTATGCATTGTATTGAAGAGCCTCATTG
GACCTTATAAATATGTATTATTACTATGTGTTAATTAAAATACATTTTAAAAGAAGACAGAATTGGATAGTGAGTT
GTAGGCTGGGTCAGAAACATTCCTTTATGTAATT**CTCCCTTCTGCAAGTATGTTAAGTCTACCTCATCTACCACAGA**
CTGAGTTACTTAAATGTTATTCCAGCAGGGGCGCCAAATCATCAGGAATGCCTCTGAGGCAGTCTCGTTGGATGGG
AGAAGAAACTAATCTCTGATGCTTCTTGAGTGTGTGTGTGTGTGTGTGTGTGTGTGTGTGTATGTGTGATAGGTCTG
GTTAGTATATCTGTGTGTGATTTGTGTATGTGTCTCATATGTGTGTGGTTGTGTGTATCATGTGTAAATGTGTATGT
GTATTGTATATGTGTGTACATATGTGCGTGCTGTATGTGCAACGTGCATATGTATGTGTGCACACGTGCTCGTGCAC

Line Δ2392/CDE
sgRNA used: C2/3, D2/3, E4
Deletion size: 2,442/4/1165 bp respectively

GTATACCTCACACATGCACACACACATGCACACACACACACACAGAGACAAGTCTTAATAAATATTAGTAAGTTATA
AATATTAACAAATACACATGAATAAAACATTAACAACATTGATATGTTTATATTTTATGTTTATTTAAATATAATAA
ATAGATAAATAAATAGTAAATGTTTTCTGTGGCTGCTCAATGGCTCATGGCTGGTCCTTAGATGGAGAGATCAAGAC
TGCCTTGGTGAAGTGAAGATTGAGAAAAAGTGAATGAAGCCACAGCAACACGAGGAAAAGATTGGCATCAAAAG**TTG**
AAGATGGTGGAAATTATGCCACTGGGAAGCAGCATATCTGCAGGTGGCTGGCTCAGTGGTTAGAAGCTCTGGCTGCT
TTTGCACAGGACCTTGGCTTGGTCCCCAGCACCCACATGGTGGCTTACAACTATCTGTAACTCCAGTTCCTGGGGAA
CCACCAGTTGGTTCTTCTAGTCTCTTGCAGAAAGCCTGTATCATGATCCTAGAACACCAGTACAGGCTAGACCCAGC
ACATTCTGATTTCCAGTGGATGTTAGAAGGTGAAAGGGGCTGGAGAGGTGGCTTCTTGTCCAGAGGAACGGGTTTGA
TTCCCAACACTTACATGGAGGTATCCATGGGTTCCAGAGGATCCACTGCCCCTTTCTGGCCTCTGAGGGCACCAGGC
AAAACACCCATACATGTTAAAAAAAAAAAAAAAAGAAAGAAAAGAAAAAGAAAAAAAAAAGAAAGAGAAAAAGACTCA
GGAGAAGACGAGGACCGTAGAACCCCTGTGTAGTCTGGAGTCAGTGAAGCTAGAAGCTAGAAGCTAGAAGCTAGAAG
CTAGAAGCTAGAAGCTAGAAGCTAGAAGCTAGAAGCTAGAAGCCAGAAAGTTCCCTGGATAGGGTTGTGTGTGTGTG
TGTGTGAATCCCAGAGACCATTCTCCAGCCCACTATTTAGACACTGCTGTTAGTACACTTGGGCATCAGAGTCTGCC
CTCAGTCAGCGCCTCTCCTCAGTAAACCAACCTATAGGGCCAACTTTAACAAAGGTAGCCACAATGGGAAGGACAAA
GAGACCCAGCAACCCTTCCCTCCCCAGGTCCATCCTCAGGGACTGAAGCTTGAGCCTCTGGCCAACAGTGAACACAT
CTGCGCAGTTCCCTAGAAAGAATGGACCCGCCCACCATCAACTTTCCTTTGCTTCCTGGTATGAGAATAAACAAAGA
CCATTCAAATGAATACACCTCCATCCAGTGACAGAGTCCCAGACAGCCGGGAGAGATGACAGCTGACAGGTCCTAAG
GGAACCAGAACCGCACCCCCCCCCCATAGCCCCCACCCCCAGCTGCTTGGGGAGGCTGGAAGCAGTCTTCTCTGATT
GGCCTAGAAAACAGGAAGCTGTTAGTTACCGGTCAAGCTCTGATTACTTGGGGTGGAGGAGTTGTTTGGCTTCATGC
TCCCTGTTATATTGACGGTGTGGCCTCATGCAAGTGTGCCTTCGGAGCCTGTGACTGGCCTGGGTGGAATCCCCTTC
TCAGGCATGTTCTGAACATGGCTTGCATAATAGACAGGCTCTCCCTGGGTTCTAGTAAGTCAGAAGACAGCCAGTTT
GGATCAGCCAGTTTGGCTTCTTTGGGAACCATCTAGAAGCCAAGCTGCTGGCGAGATGAGAAAGACAGGAACGCGGA
GGGCATCTTTAGCTTGCAACCCTGGAGAAGAGCTGCTGTTTCCTGCCTCCTTGTTGGGCAGGCCCAGTAAAGCAGGG
CAGAAGAAGTCAGGTCCGCTCATACCATGGCCATCTTCTTGCTGGATAGCTCTTGCCAGGTGTTTCTTCTGCTGAGA
ATGATGAGCGGCCCTGACACCTCAAAGCCGCTTTGCTCCTTTGCACATGGATCTCAGAAAAAGCCTCTGAGTTTCCC
ATATGAACTCAGGAGACCCAACCCAACCCAACCCAACCCAACCCAACCCAACCCAACCCAACCCAACAAACACCCAA
ACCGAGAGAAATAACAAAACGGCTCACAGCTCCCTCCACTGGCCCATATGAGTTACTGCAGCTGCTTTATCAAAAAA
CAGCTTCTGGGTCCACAGCAGAGCCAGGCTTTCTTCCAACCTTTCTAGGTCAAAGCCAAGTCCTGAGCTCCCAGTTC
TTTTCCTTGCTTTCTCCACGATACATGTCATCCCTGTTGACCCAGCTTGTTCAGCCACACCGGTTTCCTGATGGCCC
TCTCCTGCAGGAACCTCTCGCTGAGGTCAAGTGACTACATCTTTGTGGCAGTTTTTTTTTTTTTTTTTTTTTTTTT
TTTTTGTTGAGAATATAGATGAGATTGTGTGAATATCCTTGTTCTACCGAAAGCTGTGTGCATTTTAACCAATGCTG
GTGGCTCTCAGCTCACCCCTCCACACTCTGGACTGTTCTCATTAGCTTTGTTCCCTTCTTCTGAGTATTTGCATGAC
TCCACTTCACACCTGGTGAGAATTTGGTGTTTCTGGGCTCTGGCCCCGTTGGAACCAAAGCTGCTTCTCTTTCTAGG
TTCCTCTGGAGCTGACAGTACAGTTTGTCACCAGCTCATGGGGCCCCCTAGCTTGAGAGGTGCAGCTGGGGGGACTG
CCTTTTGATCTACTTCTGCTTTAGAAATATATTTATATGTGTGTGAGTGTTGTGTGTGTGCAAGCGTATATGAGTGT
GTATGTGTATTTGTGTGAGTGTGTGCATGTGTATGTGTGAGTGTGTAGGTATGTGAGTATGTATATGTGTGTGTATG
AAAGTGTATGTGTGATTGTATTTGTGTGTGAGTGTGTATATGTGTGTGAGTGTGTGTATGTGTGTATGAATGTGTAT
GTGTGTATATGTGTATATGTGATTGTATTTGTGTGTGAGTGTGTATACGTGTGTATATGAATGTGTATGTGTGTGTG
AGTGTGTGTATGTGTGTGTGAGTGTGTATGTGTGTGTGTGTATGTGTATGTGATTATATTTGTGTGTGTATATGTGA
TTGTATTTGTGTGTGAGTGTGTATATGTGTGTATGTGTGTGAGTGTGTGT

CTTCCTGGAACTCACTCTGTAGACCAGGCTGGCCTCAAACCCAGAAACCCACCTGCCTCTGCCTCCCAAGTGCTGGG
ATTAAAGGTGTGTGCCACCACTGCCCGGCCCAGGTTTGTTTTTCAGGACCCTCTTGGTGGCTCACAACTTTCTGGAA
CTCATAGTTGCAAGGGGATCTGACATCC**TCTT**CTGGTGGGCACAGGTACTGCATGCATGTGGTACACTGTCATACAT
GTAGACAAATCAACCAGATACATAAGTTAGAAATGAAGAAACTGGGCTGGTGAGATGGCTCAGTGGGTAAGAGTACC
CGACTGCTCTTCCGAAGGTCTGAAGTTCAAATCCCAGC

TTACTGTGATTGACAGTGATTTAAAATGAATATTTTAATGTAGTCAGTAGAGTGGGTTGTAAAGGTTCTCAACAGAA
AGAAAGGCTGTCTGTCTGAGACAATAGGCCAGTTTCCCTGACTTGGTCATTATGCATTGTATTGAAGAGCCTCATTG
GACCTTATAAATATGTATTATTACTATGTGTTAATTAAAATACATTTTAAAAAGAAGACAGAATTGGATAGTGAGTT
GTAGGCTGGGTCA**GAAACATTCCTTTATGTAATTCTCCCTTCTGCAAGTATGTTAAGTCTACCTCATCTACCACAGA**
CTGAGTTACTTAAATGTTATTCCAGCAGGGGGCGCCAAATCATCAGGAATGCCTCTGAGGCAGTCTCGTTGGATGGG
AGAAGAAACTAATCTCTGATGCTTCTTGAGTGTGTGTGTGTGTGTGTGTGTGTGTGTGTATGTGTGATAGGTCTG
GTTAGTATATCTGTGTGTGATTTGTGTATGTGTCTCATATGTGTGTGGTTGTGTGTATCATGTGTAAATGTGTATGT
GTATTGTATATGTGTGTACATATGTGCGTGCTGTATGTGCAACGTGCATATGTATGTGTGCACACGTGCTCGTGCAC
ACATTGCATGCACATGTGCCTCTGTGTGCAGCTATGTGGCAGAAGTGTCCAGTTCAGGGAGAGGGCCAGGCAAGATA
CTGTGCATTTCTGTCAGGTGGAAATGGGGTGCTATCTGATCACAATGTCCTCTTAGAAGCCGATCGTGGAGGAGAAC
TCCTTAAACATTTAAGAGGAAGAGTAATTGGCTGGCCACAGTGCCTTGAATTGGGAAGTTGGTCTAAAGGCTGGTGA
ACGATGTCGGGGTAAGAGAACCCTAGAGCACAAGATGCAGGCTCAAGACGGCTCAGCCTGACGAGTCTGAGAATATA
GATGCGTGCACACATGTGTGGGAGGAGTGGCCATTCCCGTTGTAGATGGAAAAACAGCAAAGATCAACAGCAACCTT
GGCAACCTCACTGGTGGGCTGAGAGGTGTACGAGGGATGCAGTAGCTATTGTGGGGCCTCCTGACCCACGGTGGCTC
CAGGAGTTCCAAGGCTCATCAATTAATATGCTCTTGTCCTCAGAGTGATGGCCCCAGCAGATGGCCAGCATTTCTGA
CATTTACAGAGCTCCAGCTCACGGACATTCTGACTTTAGGCACCAGCCGAGTGGTCTCGCTCCAGCCCCATGCTTCA
CTTCCTCAGGTCCTGTTCTGCCAGGACAACCCATCTTCATCTCTAGTGTGTTCATCACCTCACCTGCCGAAAACCCT
AGGGCAGGGGAATTCGTCCTGCTCTAGTGTGTGGGGCAGGTTTGGTTGGCATTGCTCAAGCCCTATCCTCAACCATG
TTCCTGTCGGGTCTGGACCATCAGGTAAGCTCTCTACCCTAGGGATGCCGTACACAGCTTGGGTTTTCCCCCAAGCC
AGCCCTACCGAGTGCTGAGCAAGGAAATGAGTGCTCCATGTTATTAATCACTTACTGCCGCTGAGCCTGAGGGTCAC
AGCCATCAGAGACGGATGCCTGCTTCAGGCCAGGAGTCAGGATCTTGTGGAAGA

Supplementary Note 2: eRNA associated with S3 was identified and characterized using 5' and 3' RACE

5′   TCTCTCTCTCTCTCTCTCTCTCTCTCTCTCTCTCTCTCTGGAAGAGCAGTCAGTTCTCTTAACCACTAACCCA
TCTCCAGCCCAAGGCTACTGCCATTTTGAAGCATTTTCTTAGCTTCTAAAGAATCAGTTTCAGAGCGTTGCATGGTG
GCTCACACCTTTGATCCCAGCAGTAGGAGTCAAAGGCAGACAGATCTGTGGGTTCCAGGCCAGCCTGGTTTATGTAG
TGAGCTCTAGGCCAGTGAGGGCTATATCACATCGGTTTTAGGGGGCAACTGTGGGACATGAACCAGAACCCTTCCTC
TCTGCACTCTGTGTGGACAGAGGGGAGAGCAGAAGTCTGTTCTTGTGTCCCAGTACTCAAGGGAACCTAGCCTTGAG
GCCCAGGGTGACACTGGTCAGTTGAGGACATGTGTTCCTGTGTGGTGGGGAAGTGTAGGGAGGACAGAGAGGCCCTG
GCTGAACAAGGAGTTCATGAGGTGCACACATCTCCTCAGAGCCTGCAGGCCAGGTCGGGAAGCCAGAAGGTTCTAGG
AATGAAGACATTACTCTGAATTCACCAGGCCCAGCGGCAAGGGGCATGGGGTTCTTATGCCCTGGGGTGTTCTGGTT
CTTTGGCCTGCCAGTCTTGAGCCTGTGTGCTTCCAGTATGCGTAGGCTCTTTCCTGCCCCATCAACTCTGCTCGGCT
AGGGTGGGAAAAGGACACCAGGGTTTTGGTAGTACACACACTGCACTTTTTGTCCTATGGGGTCAGCTGTGCTGGAA
TTTTCCCTAAAAACCTGAGCAGTGGACAGGGTGGAAGAGGAGGACAGGGTGATGGAGGAGGACAGGCTGCCTGCCCC
ACGAGCTTGCTTCTTTCTTGTAGTGCTTTTATGGCTCCTGGATGAACAGCTTGCCCCATTCTTGGCCATGGTGCTTA
GGAATTCTGAGTGAGAGTCTCTCCCTCTTCAGTGAAGTCACGACTACATGTCATCCTTGTCACTGTGCTATGTGCAG
ACCTTAGTCATAGGCCTTGCCTGCTCATTCTGAGGCTCTCCCTACACCCCCTCATATCCCTCATCATATCCGTATAC
AGTGTGGTCTCTCCTTCCCCAAAGCTCCTTGGCCTGTCCCCTGCTCAGCAGCACCAAGGGTAATTGTGTTAGACACC
CCAGCTGGCACCGGCTGCTCTAAGCAACAGCAAGGAGGTAGCAGCTGCATGGCTGCTCTACTGCACTATGTGGCTCT
GGCATGTTCCCTTGTGGCTCGTTAGCTAGCCCATCTGTGTTGGGTCCAGCGGACGGGGTGCAGCCTCCAGAGGTGCC
CCCAGGAGGGAGTGATTGTTGGCACAGAGTCTGGGGTCACCATGTTTAAGTGAGAAGGCAGAAGTGCTGAGAAGGGG
TGAGGTGTTGAGCCCCTTTGGTGAGAAGGAGGAGGGCCTGGCCTCTCCTCTGATGAGAGGAGAAGTTTGACCTGCTA
CTCCCTCCTTCAGAAACCTGCCAGGAGGGTCTCCATTGGAACAGGGACTGTGGAGAATTAGCAGGTGATAGAGAAGT
GGTTTCTAAACAGGCCCAGGAAACCCTTTACAGTCTAGGGGGATTTTTTTCAGTCATGCTGTGATTTAGAGGTAGAC
TTGGGACAAGCTCGCAGGTTTGTGGATCTTGAAGGACAGTAGGGGTCAGAGCATCAAGTTGATCCCCACCTTGGAAA
AGGGGCCCACACTTCCTCTTTCTACCACTCCTTAGGCACAGGGCAAGGAGCTCCTGGGCTCAACCTTGGGTCTGAGA
GGAGATGCCTGGGAGGAGTCCCTGAGTTTTCCCTCAAGTAGAGATTGGGTTAGGTGTTCCAGTCTGCTGAGAATGTT
CAGGGCCTTGGCCACCCTCTACAGAAGCAGCTCACTGTGCTTGATACATAAACACACATGGGTCAACAGTTAAGAGC
ACTTGTTGCTCTTGCTGAAGATGCAGGCTTGATTCCCAGAGCCCACATAGTAGCCGAGGATGGCCCTAGGTCCAGTT
CTAGGGCACCTGCCTCCTCCTTCTAGTCT(AAAAAAAA)   3′

**Supplementary Table 1:** sgRNA used to generate CRISPR/Cas9 knockout mice	
sgRNA	Sequences
C1	5′-GGTAACTCATATGGGCCAGTGG-3′
C2	5′-TGAGTTTCCCATATGAACTCAGG-3′
C3	5′-TCCTGATGGCCCTCTCCTGCAGG-3′
D1	5′-CCTCTTCTGGTGGGCACAGGTAC-3′
E1	5′-GACTTAACATACTTGCAGAAGGG-3′
E2	5′-AACTCAGTCTGTGGTAGATGAGG-3′
E3	5′-CTGAGGCAGTCTCGTTGGATGGG-3′
E4	5′-GGAACTCAGTCTGTGGTAGATG-3′
	green = NGG

Supplementary Table 2: Primers

Name	Sequence
Site A/B Promoter Proximal LoxP PCR F	5'-AGCTCTACTCCCCAGCACAAG-3'
Site A/B Promoter Proximal LoxP PCR R	5'-TGGGAATGGGTTCATTTTCTCTGC-3'
Site A/B Null Allele PCR F	5'-CCAGGAAGTGGGTATGGGTA-3'
Site A/B Null Allele PCR/ R	5'-GGGGAACCCCAGGAGAGC-3'
Site A/B Null Allele Sanger F	5'-AATTGTTCTGGGCTGGTGAG-3'
Site A/B Null Allele Sanger R	5'-GCCTAGGGAATCAGGGTTCT-3'
Site C PCR F	5’-GCTGGATAGCTCTTGCCAGGTG-3’
Site C PCR R	5’-GAGGTTCCTGCAGGAGAGGG-3’
Site C Sanger F	5’-GAGAATGATGAGCGGCCCTG-3’
Site C Sanger R	5’-GGTGTGGCTGAACAAGCTGGG-3’
Site D PCR F	5’-GCTTGAAGGCAGATCTTTTGA-3’
Site D PCR R	5’-TCTATGCCATGTCCCTCCTC-3’
Site D Sanger F	5’-GGCACACACACATGCCCAGG-3’
Site D Sanger R	5’-TACAGGTGGTTGTGAGCCACC-3’
Site E PCR F	5’-CCCTGACTTGGTCATTATGCATTG-3’
Site E PCR R	5’-CACGAGCACGTGTGCACAC-3’
Site E Sanger F	5’-GTATTGAAGAGCCTCATTGG-3’
Site E Sanger R	5’-GTTTCTTCTCCCATCCAACG-3’
2392A F	5'-GCTCAATGGCTCATGGCTGGTCC -3'
2392A R	5'-CCAGCTGCACCTCTCAAGCTAGG-3'
